# Distal Femoral Oblique Fracture in a Young Male Soldier: Case Report of an Unusual Presentation

**DOI:** 10.1097/MD.0000000000003776

**Published:** 2016-06-03

**Authors:** David Naji Cohen, Hesham Al Khateeb, Mohammed Safwat

**Affiliations:** From the Royal College of Surgeons in Ireland—Bahrain (DNC); and King Hamad University Hospital (HAK, MS), Kingdom of Bahrain.

## Abstract

Here, we report a case of a distal femoral fracture in a 23-year-old male army cadet who presented to the Accident and Emergency department following a twisting injury while participating in a routine military marching exercise. A pathological fracture was considered but this suspicion was put to rest following thorough investigations, leaving only a diagnosis of a nontraumatic spontaneous femoral fracture.

To our knowledge, there have been no reported cases of distal femoral fractures associated with nontraumatic military exercises, with the majority of injuries instead related to stress fractures. A vigilant literature search yielded no cases of similar injury nature, which is the primary reason we believe that those interested in orthopaedics or military doctors would find themselves drawn to this case.

The patient presented with severe pain in his left thigh and on examination there was a deformity of his left thigh.

In terms of investigations, a bone profile, plain film radiographs, C-reactive protein, erythrocyte sedimentation rate, and tumor markers were all preformed and proved unremarkable. The definitive treatment was by open reduction and internal fixation.

Femoral fractures often require significant amounts of force, particularly in young, healthy individuals. Generally, these injuries in this demographic follow high-energy traumas, with the lion's share occurring following a road traffic accident or other high-speed impact. More often than not, the treatment is surgical. Given the extraordinary manner of this such, one must be attentive and exhaustive in their investigation of such presentations.

## INTRODUCTION

While military personnel are no strangers to distal femoral fractures sustained during service or training, these can most often be attributed to stress. Previous literature has reported on stress fractures among military trainees,^1^ but the atypical history and severity of this gentleman's fracture has prompted much interest and discussion among the department.

Kolmert and Wulff^2^ spoke at length about the demographics commonly associated with distal femoral fractures. The strongest correlation is with increasing age, where 84% of patients were above the age of 50. Meanwhile, fractures seen in younger patients were more likely to be in a male. All fractures in this particular study had been caused by trauma, one-third by severe and two-thirds by moderate.^2^ Fractures following severe trauma were strongly associated with those in the younger age group. This is a particularly remarkable finding given the history of this case. Other risk factors associated with distal femoral fractures include disease-related impaired function in 1 or both legs, and a previous fracture in the same leg.

## CASE REPORT

We present a 23-year-old male patient who recently enlisted in the army. He presented to the Accident and Emergency department following a twisting injury while exercising.

The patient was oriented to person, place, and time. Analgesia was administered in the form of tramadol. The patient complained of severe pain at the level of and superior to his left knee and an inability to actively move his knee. This started immediately following an injury sustained while walking backward and turning left. He denied any falls, previous fractures, or previous medical and surgical history.

On examination, the orthopaedics team on call found marked swelling around the left knee and there was an obvious deformity. Upon palpation, the thigh was found to be tender and painful proximal to the knee. Neurovasculature was all revealed to be in tact.

AP and lateral plain film radiographs of the left pelvis, hip, femur, and knee demonstrated a distal oblique fracture of the left femur, which was partially displaced posteriorly and medially. A decision was made to admit the patient for open reduction and internal fixation to be performed the following day. In the meantime, a backslab was applied and plain film radiographs were repeated. Once on the ward, consent for surgery and anesthetic was obtained and the patient was maintained on nil per os.

The patient was then moved to the operating theater where an open reduction and internal fixation was performed. Anatomical reduction was achieved using an AO Synthes locking plate and screw construct through a direct lateral vastus sparing approach. The decision to plate the fracture was taken because of the patient's extremely narrow medulla in the femur.

The patient had an uneventful postoperative recovery and was mobilized nonweight bearing on crutches on day one. Postoperative radiographs confirmed anatomical reduction and the patient was allowed passive range of movement on day three.

At latest follow-up, the patient achieved a range of movement of 0° to 100° and the fracture was uniting radiologically.

**FIGURE 1:**
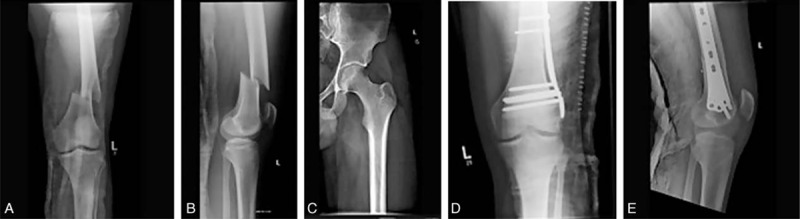
Plain film radiograph AP field preoperative (A); plain film radiograph lateral field preoperative (B); plain film radiograph of medullary canal (C); plain film radiograph AP field postoperative (D); plain film radiograph lateral field postoperative (E).

The narrow medulla, as well as the fracture both pre and postoperatively, can be well visualized in Figure 1.

## DISCUSSION

Our literature search has yielded no similar cases of such a fracture following minor biomechanical forces in a young and otherwise healthy male soldier.

In this 23-year-old soldier's case, the patient denied any history of trauma or road traffic accident and this was confirmed on physical examination by the absence of lacerations, abrasions, or bruises over the whole body and specifically, at the fracture site. Clinical and radiographical evaluation showed no evidence of fractures elsewhere. Using the so-called “Surgical Sieve” the potential causes of the fracture were systematically considered. Possible likely causes could include metabolic, inflammatory, neoplastic, degenerative, infection, and traumatic. Understandably, the seemingly trivial nature of the forces that would have been acting upon the femur while marching was a source of worry for the orthopaedic team. Suspicions of a pathological fracture were raised given the atypical history of the case.

However, the combinations of the patient's own history, his family history, and all investigations have been unremarkable and have not revealed any sinister underlying pathology.

Supracondylar distal femoral fractures are shown to have a bimodal age distribution.^3^ These fractures are usually seen in 2 groups of patients. In younger patients, comminuted, often intrarticular fractures, are most often caused by road traffic accidents and sport injuries and are best treated with anatomical reduction and internal fixation. In contrast, distal femoral fractures in elderly patients are often simple and caused by minor trauma. The osteoporotic bone and patient's comorbidities and poor mobility make treatment difficult.^4^

In their 1985 study of Stress Fractures in Military recruits, Milgrom et al^5^ stated, “in any population subjected to strenuous physical training, incidence of stress fractures may reach very high proportions.” They also stated that as many as “sixty-nine percent of the femoral stress factors were asymptomatic.” The bizarre nature of our patient's fracture may have some relevance here; given that he had only been in the army for 3 months, the level of physical activity he had been exposed to in this time may very well be far higher than he has ever undertaken before. While far-fetched, this may be a contributing factor to his femur being weakened to the point of fracturing following relatively negligible twisting forces.

Niva et al,^6^ in their study regarding fatigue injuries of the femur, documented that up to a quarter of all bone fatigue injuries are seen in military recruits. This strong link between femoral fatigue fractures and military service serves to reinforce the extraordinary nature of our case. One would not be unwise to put a stress-related femoral fracture high on the list of differential diagnoses when assessing a young soldier.

Sickle cell disease is a prevalent condition in the Middle East. Akinyoola, Orimolade, and Yusuf carried out a study,^7^ published in 2008, on the causes of pathological fractures in Nigerian children. This study demonstrated that just over 1 quarter of the pathological fractures could be attributed to Sickle cell disease. Therefore, it was perhaps reasonable for the doctors here to add this to the list of differential diagnoses with regards to the cause. This was of course found not to be the case.

The possibility of Osteogenesis Imperfecta was considered as well. Nicolaou et al informed us that “decreased bone density, abnormal mineralization of the extracellular matrix, disuse osteoporosis, and altered bone geometry lead in most cases, to transverse or short oblique” fractures. The oblique pattern of our patient's fracture made Osteogenesis Imperfecta a very realistic cause of his fracture. However though, a lack of family and personal history of repeated fractures, as well as the unremarkable bone profile, allowed us to rule out this as a contributing factor.

Osteomyelitis was recognized as a possible cause; however, a lack of previous systemic symptoms and the normal values for C-reactive protein and erythrocyte sedimentation rate helped us to rule this out.

Last, a primary neoplasm was discussed as a feasible underlying pathology. However, the denial of constitutional and direct symptoms, the lack of radiographical evidence, and the absence of elevated tumor markers on the bone profile all negated the likelihood of this pathology.

With regards to traumatic injury causing this fracture, we were not convinced of this possibility from the start because of the history of the presenting complaint. On website of the Royal College of Surgeons, Edinburgh, Rastogi found the forces necessary to fracture a femur to be enormous; the average velocity change in a road traffic accident had to be “42 km/h (26 mph).” On the same page, Kyle estimates that the bending moment acting on a femur had to be about 250 Nm for it to fracture.^8^

All of these possibilities had to be ruled out one by one. The spontaneous and nontraumatic nature of this fracture are what we believe make this case such a unique and noteworthy one. We believe that given the frugality of our literature search, this will be a case that is of great interest to the Orthopaedics community here in Bahrain, the Middle East, and further afield.

## CONCLUSION

In summary, after thorough investigations were carried out, all of our initial possibilities had been systematically ruled out and we were left with the sole diagnosis of a spontaneous and nontraumatic distal femoral fracture in an otherwise healthy young man. A meticulous and comprehensive literature search has revealed no cases of a comparable nature; thus those involved in orthopaedics, emergency medicine or military doctors may take interest in this case.
